# Does the Need for Control Hinder Sense of Presence in Virtual Reality?

**DOI:** 10.5334/pb.1324

**Published:** 2025-05-12

**Authors:** Romane Michaux, Céline Stassart, Aurélie Wagener

**Affiliations:** 1 Research Unit for a life-Course perspective on Health and Education (RUCHE), Health Psychology, Department of Psychology, University of Liege, Liege, Belgium

**Keywords:** Sense of presence, Virtual reality, Need for control

## Abstract

Virtual reality (VR) has witnessed significant growth in the mental health field. However, clinical observations reveal substantial variability across individuals’ responses to VR. This diversity could be attributed to differences in the sense of presence, a key factor in VR’s efficacy. Understanding the influence of personality traits on shaping the sense of presence in VR is essential, as it holds the potential to enhance the effectiveness of VR interventions. In the current study, we investigate the potential impact of the need for control on one’s sense of presence in VR, hypothesizing that a higher need for control is associated with lower presence levels. We conducted research with 40 adults who completed questionnaires (assessing perfectionism, intolerance of uncertainty, experiential avoidance, Big Five, sense of presence, anxiety level) and engaged in a VR immersion. Results suggest that the need for control positively influences the sense of presence, which is contrary to the literature. A discussion is proposed to explore the impact of high need for control, indicating that its effects may depend on the anxiety-inducing nature of the immersion condition. We put forth an innovative theoretical model on how a strong desire for control could affect the sense of presence in different immersive conditions. Our findings warrant further investigations in this direction.

## Introduction

Virtual reality (VR) is commonly defined as a technological communication interface that enables users to actively engage in a three-dimensional computer-generated virtual world, providing opportunities for immersive interactive experiences comparable to those encountered in the physical world (Maples-Keller et al., 2017). This emerging technology is experiencing significant growth within the field of mental health, opening new avenues for patient’s care (Riva, 2022). Indeed, it proves to be effective in the treatment of various psychological disorders, such as anxiety disorders (Fernández-Álvarez, Di Lernia & Riva, 2020), post-traumatic stress disorder (Maples-Keller et al., 2017), schizophrenia, eating disorders, and addictive disorders (Grochowska, Wichniak & Jarema, 2019). Furthermore, it is regularly employed as a relaxation and emotional regulation tool (Veling et al., 2021). However, in clinical practice, each individual responds to VR differently (Coelho et al., 2006). Research has also revealed that the same immersive system can generate varied degrees in the sense of presence from one person to another (Coelho et al., 2006). The sense of presence is a subjective illusion linked to one’s perceptual and cognitive experiences (Morélot et al., 2021). More concretely, it refers to the sensation of being transported in a virtual environment, different from the actual physical localisation (Servotte et al., 2020). Experiencing a high level of presence is a key element in VR’s efficacy since it enables the generation of emotions in users which is useful to achieve the intervention’s objectives (e.g., exposure to an anxiety-provoking situation, reaching a state of relaxation) (Cerda et al., 2021; Freeman et al., 2017; Morélot et al., 2021; Sacau et al., 2005). Consequently, there is a pressing need to explore strategies for enhancing the sense of presence, as it holds the potential to optimize the effectiveness of VR interventions. Recently, Thorp and colleagues (2023) highlighted the importance of investigating the impact of stable personality traits on the sense of presence. Among these, the desire and the need for control have been little investigated in the VR field.

“Control” is a broad concept including the *desire for control* (or need for control) and the *sense of control* (or perceived control) (Moulding & Kyrios, 2007). On the one hand, the desire for control corresponds to one’s general motivation to exert a control over events (Burger & Cooper, 1979). On the other hand, the sense of control corresponds to the extent to which one perceives that he has control in a given context (Moulding & Kyrios, 2007). Exploring these two concepts concurrently is essential due to their inter-connections (Moulding & Kyrios, 2007).

Indeed, the desire for control varies among individuals (Gebhardt & Brosschot, 2002), and the resulting consequences for individuals depend on the actual available opportunities for control in the environment (Burger & Cooper, 1979; Shapiro, Schwartz & Astin, 1996; Zhang et al., 2018). When an individual with a strong desire for control faces a situation where exerting control becomes challenging, it can lead to negative psychological outcomes, including anxiety or depression (Moulding & Kyrios, 2007; Ruggiero et al., 2012; Zhang et al., 2022). As suggested by Witmer and Singer (1998) in their explanatory model of the sense of presence, the factor of control appears to have an influence on the sense of presence (Ochs, Jain & Blache, 2018). This factor refers to the control available in the virtual environment, such as degree of control, immediacy of control, anticipation, mode of control, and interaction with the physical environment. From a clinical perspective, Wallach and colleagues (2009) identified a negative correlation between avoidance behaviours and the sense of presence in a virtual environment. Specifically, the more individuals engage in avoidance behaviours during VR immersion, the lower their sense of presence, ultimately resulting in a less effective experience. Therefore, it seems compelling to investigate the impact of one’s intrinsic control on the sense of presence he may experience.

In the current study, three psychological constructs – namely perfectionism, intolerance of uncertainty, and experiential avoidance – were selected to represent the desire for control due to their documented associations with this concept in the literature. Firstly, perfectionism, is characterized by the tendency to set excessively high goals, and to consistently perceive a gap between these goals and one’s achievements, which leads to the need for control to reduce this gap (Seong, Lee & Chang, 2021). Secondly, intolerance of uncertainty is defined as the difficulty in managing unpleasant emotions associated with the perception of uncertainty, and an inability to tolerate the absence of clear and complete information, leading to an experienced loss of control (Cobos et al., 2022). Thirdly, experiential avoidance is defined as the inclination to control or avoid unwanted internal experiences (Rochefort, Baldwin & Chmielewski, 2018), such as thoughts, emotions, bodily sensations, images, memories, or behavioural dispositions (Berghoff et al., 2018).

The objective of this study was to investigate the potential impact of the need for control on one’s sense of presence in VR. It was hypothesized that higher levels in the need for control will be associated with lower levels of presence.

## Materials and Methods

### Participants

The study sample was composed of 40 adults aged between 22 and 65 years (M = 35, SD = 14.45). The inclusion criteria were being at least 18 years old and speaking French fluently. Three non-inclusion criteria were considered: participants should not have a history of recurrent headaches, and/or photosensitive epilepsy, and/or motion sickness (Malbos & Oppenheimer, 2020).

A power analysis was conducted using the G*Power software to determine the required sample size for correlations, which was computed a priori (0.5 effect size, 0.05 Type 1 error rate, 0.95 power). The results indicate that a sample size of at least 34 participants was required. Therefore, a sample of 40 participants was constituted. Participants were recruited with announcements on social media.

The protocol received approval from the institutional ethics committee (approval number: 2223–044). All participants willingly participated and provided informed consent by signing a document ensuring their anonymity. They were also informed of their right to discontinue their participation at any point without the need to provide a justification.

### Design

The experimental design was structured into three phases: two questionnaires’ sessions interspersed with one immersive session. All steps occurred on the same day. The first phase, conducted just before the immersive session, involved the assessment of socio-demographic data, big five personality traits, the current state of anxiety and targeted variables (i.e., perfectionism, avoidance, and intolerance of uncertainty). Subsequently, participants were immersed, using the Oculus Quest 2 headset, for 12 minutes within the virtual environment “Nature Trek VR”, developed by John Carline in 2017 (Nature Treks VR, n.d.). This program was selected for its ability to create a neutral yet interactive virtual environment, as well as for its accessibility and availability. Although the design of the environment was created with the intention of inducing relaxation, this was not the primary objective in the current study. To minimize the relaxation effects, the background music was disabled, leaving only ambient forest and wildlife sounds. To enhance participants’ interest, they were given the choice among three pre-selected environments: a springtime, an autumnal, and a wintry setting. During the immersion, participants had the opportunity to interactively explore a natural landscape within a forest inhabited by various animals. Participants were able to engage with their surroundings in meaningful ways, such as grabbing branches, growing flowers and trees, and modifying environmental variables like the time of day or weather. Immediately after the immersion, the sense of presence and the participant’s current state of anxiety were assessed.

### Measures

#### Socio-demographic Questionnaire

Participants were invited to indicate their gender and age.

#### Desire for Control

Three variables were selected to assess the desire for control: perfectionism, experiential avoidance and intolerance of uncertainty. No dedicated Desire for Control scales were used.

##### Multidimensional Perfectionism Scale

The Multidimensional Perfectionism scale (FMPS) (Stöber, 1998) measures one’s degree of perfectionism. It comprises 35 items divided into four dimensions as suggested by Stöber: concerns over mistakes and doubts (CMD), personal standards (PS), parental expectations and criticism (PEC), and organization (O) (Stöber, 1998). Participants were asked to rate each item on a Likert-type scale ranging from 0 “strongly disagree” to 5 “strongly agree”. This questionnaire has a good internal consistency (Stöber, 1998) and satisfactory validity (Hewitt et al., 1991).

##### Brief Multidimensional Experiential Avoidance Questionnaire

The Brief Multidimensional Experiential Avoidance Questionnaire (MEAQ-B) (Gamez et al., 2014) is derived from the 62-item Multidimensional Experiential Avoidance Questionnaire (Gamez et al., 2014). The brief version comprises 15 items evaluating explicit avoidance behaviours, distress-related attitudes and beliefs, implicit avoidance behaviours, and the subject’s ability to effectively respond to distressing situations. Each item is rated on a Likert-type scale from 1 “not at all agree” to 6 “completely agree”. Cronbach’s alpha exceeds 0.80, indicating satisfactory internal consistency. The validity of the questionnaire was satisfactory (Gamez et al., 2014).

##### Intolerance of Uncertainty Scale

The Intolerance of Uncertainty Scale (IUS) (Sexton & Dugas, 2009) assesses emotional, cognitive, and behavioural responses when individuals are confronted with uncertainty. It consists of 27 items placed on a Likert-type scale ranging from 1 “not at all like me” to 5 “exactly like me”. Its internal consistency is excellent, with a Cronbach’s alpha of 0.91, as well as validity indices (Freeston et al., 1994).

#### Big Five Inventory

The Big Five Inventory measures five major personality traits: openness to experience (O), conscientiousness (C), extraversion (E), agreeableness (A), and neuroticism (N) (Plaisant et al., 2010). It comprises 45 items on a Likert-type scale ranging from 1 “strongly disapprove” to 5 “strongly approve”. The internal consistency of its different dimensions’ averages at 0.79, and the validity of the scale was satisfactory (Plaisant et al., 2010).

#### State Anxiety Level

To assess the level of state anxiety, participants were instructed to indicate their anxiety levels on a visual analogue scale, in both pre- and post-immersive phases by adjusting a slider ranging from 0 “not stressed” to 100 “very stressed”.

#### Cybersickness Questionnaire

The Simulator Sickness questionnaire (SSQ) investigates the potential negative sensations experienced by users during immersion, the cybersickness symptoms (Kennedy et al., 1993). Cybersickness is characterized as a physical condition in which users experience symptoms akin motion sickness (Rebenitsch & Owen, 2020), such as nausea, dizziness, cold sweats, or vomiting (Kennedy, Drexler & Kennedy, 2010), induced by virtual stimuli. Consisting of 16 items rated on a Likert-type scale ranging from 0 (“not at all”) to 3 (“severely”), the SSQ evaluates two distinct scales: oculomotor symptoms and nausea-type symptoms. It demonstrated good internal consistency, as evidenced by a Cronbach’s alpha coefficient of 0.86 (Bouchard et al., 2008). This questionnaire was assessed in both pre- and post-immersive phases to monitor potential symptoms that may have been present prior to the immersive experience.

#### Presence Questionnaire

This questionnaire measures the sense of presence experienced by the participants. It consists of 16 items on a Likert-type scale ranging from 1 “strongly disagree” to 7 “strongly agree”. In addition to a global score, the questionnaire includes four subscales: place illusion (i.e., the illusion of being in the virtual place), plausibility illusion (i.e., the illusion that the virtual scenario is happening), co-presence illusion (i.e., the illusion that we share the virtual environments with others), and social presence illusion (i.e., the illusion that we are psychologically related to others in the environment) (Della Libera et al., 2023). The psychometric properties (internal consistency and validity) of this questionnaire have been documented by Heck, Wagener, and Simon (2021).

#### Data analyses

All statistical analyses were conducted using SAS 9.4 software. The statistical significance threshold considered was 0.05. Correlations were conducted between sense of presence and desire for control, as well as between sense of presence and Big Five personality traits. Finally, a comparison of state anxiety levels between pre- and post-immersive phases was performed through Wilcoxon signed-rank test.

## Results

### Socio-demographic data

Socio-demographic data are presented in Table 1.

**Table 1:** Socio-demographic characteristics.


		M (SD)	MIN-MAX

Age		35 (14.45)	22–65

		n (%)	

Gender	Women	19 (47.5)	

	Men	20 (50)	

	“Other”	1 (2.5)	


### Correlations between Desire for Control and Sense of Presence

Pearson correlations revealed significant positive associations between experiential avoidance and two indicators of the sense of presence: the global sense of presence and social presence illusion, and significant positive correlation between co-presence illusion and personal standards (Table 2).

**Table 2:** Correlations between Desire for Control and Sense of Presence.


	PERFECTIONISM^1^					EXPERIENTIAL AVOIDANCE	INTOLERANCE OF UNCERTAINTY

	GLOBAL	CMD	PEC	PS	O		

Global Sense of Presence	0.15	–0.01	0.04	0.21	0.30	0.35*	0.21

Place illusion	–0.05	–0.11	–0.14	0.09	0.23	0.01	–0.14

Plausibility illusion	0.11	–0.08	0.15	0.07	0.31	0.31	0.26

Co-presence illusion	0.29	0.12	0.11	0.39*	0.24	0.26	0.19

Social Presence illusion	0.12	0.04	0.07	0.05	0.15	0.34*	0.21


* *p* < 0.05. ^1^ CMD = concerns over mistakes and doubts, PEC = Parental expectations and criticism, PS = Personal standards, O = Organization.

### Correlations between Big Five traits and Sense of Presence

Pearson correlations highlighted a significant positive relationship between conscientiousness and plausibility illusion. Spearman correlations revealed a significant positive association between conscientiousness and spatial illusion (Table 3).

**Table 3:** Correlations between Big Five personality traits^1^ and Sense of Presence.


	O	C	E	A	N

Global Sense of Presence	0.01	0.29	–0.03	–0.09	–0.04

Place illusion	0.29	0.37*	0.13	0.12	–0.22

Plausibility illusion	–0.19	0.37*	–0.02	–0.13	–0.07

Co-presence illusion	0.04	0.10	–0.01	0.0004	0.15

Social Presence illusion	–0.04	0.09	0.03	–0.16	–0.06


* *p* < 0.05.

### Comparisons of State Anxiety Levels and Cybersickness Symptoms between pre- and post-immersive phases

The Wilcoxon signed-rank test revealed a significant reduction in anxiety level experienced by participants after the immersive phase, as well as a significant increase of cybersickness symptoms, including both oculomotor and nausea-type symptoms (Table 4).

**Table 4:** Results of comparisons of State Anxiety Levels and Cybersickness Symptoms between pre- and post-immersive phases through Wilcoxon signed-rank test.


	PRE-IMMERSION		POST-IMMERSION		S	*P*
	
	M	SD	M	SD		

State Anxiety Levels (%)	18.65	26.92	5.90	10.32	–165.5	<0.0001

Global cybersickness	6.53	6.65	21.23	5.59	391	<0.0001

Oculo-motor symptoms	4.53	3.78	10.30	3.57	408	<0.0001

Nausea-type symptoms	2	3.38	10.93	2.57	406.5	<0.0001


## Discussion

This study delved into the potential influence of the need for control on one’s sense of presence in VR. Our initial hypothesis posited that individuals with a greater need for control would experience a lower sense of presence during VR immersions. Surprisingly, our results diverge from our anticipated direction and point towards the contrary trend.

Significant positive correlations were found between avoidance behaviours and the sense of presence, both in terms of global score and the illusion of social presence. These results contrast with the existing literature. Indeed, in Wallach and collaborators’ research (Wallach, Safir & Almog, 2009), avoidance behaviours were negatively associated with the sense of presence. This discrepancy may be explained by differences in how avoidance was measured. Specifically, in Wallach’s study, avoidance was observed directly during the VR immersion – for examples, participants with aviophobia avoided looking through the airplane window. In that case, avoidance reflected a *situational* expression of control. In contrast, the current study used a measure of avoidance as a *general personality trait* – reflecting a broader tendency toward avoidance and need for control, rather than moment-to-moment behaviours during immersion. We further explore this divergence from Wallach and colleagues’ (2009) findings considering the literature on the need for control.

A positive correlation was found between personal standards and the co-presence illusion, suggesting that an increased focus on self-image and self-evaluation tendencies corresponds to a heightened awareness of the significance of others’ perceptions within the environment. Two additional correlations approached significance (p = 0.05), linking the organization subscale with both the plausibility illusion and the global sense of presence. These associations suggest that increased scenario plausibility may enhance organizational facet satisfaction. With a larger sample, a clearer relationship might emerge between perfectionism and sense of presence, given the link between conscientiousness and organizational skills.

Positive associations were observed between conscientiousness and the sense of presence, specifically in terms of place and plausibility illusions. One possible explanation is that conscientious individuals tend to engage fully in VR experiences, which may enhance their sense of presence. Moreover, given the established link between conscientiousness and the need for control (Myles, Connolly & Stanulewiz, 2020), these findings support the idea that the need for control positively influences the sense of presence.

The lack of association between intolerance to uncertainty and sense of presence may be explained by how uncertainty is conceptualized. As Freeston et al. (1994) highlighted, intolerance to uncertainty is *a trait* distinct from the *emotional state* of feeling distressed due to uncertainty. It is possible that VR immersions primarily trigger emotional responses related to situational uncertainty, rather than activating the trait itself. In the present study, the virtual environment did not appear to induce distress related to uncertainty, which may explain the absence of control-related behaviours. Witmer and Singer’s model of presence (1998) introduced the idea of “*anticipation*”, suggesting that environments enabling users to predict what will happen – regardless of actual control – enhance the sense of presence. From a user-centered perspective, this implies that high uncertainty in an environment could reduce presence by causing discomfort. This perspective should be addressed in future studies.

The significant reduction in anxiety levels suggests that virtual environments maintained its relaxing properties. Although we attempted to limit these effects by removing the relaxing music and keeping only natural forest ambient sounds, immersion in a natural virtual environment alone was sufficient to reduce anxiety (Riches et al., 2021). This highlights the inherently calming nature of such immersive settings, which may have influenced the results by providing a non-anxiety-inducing context.

Overall, our results suggest that *the need for control positively influences the global sense of presence or some specific dimensions of it*. In the following, we propose a theoretical reflection – leading to a hypothetical model – to explain the relationship between control and presence, considering the anxiety level induced by the virtual environment. Burger (1985) described the need for control as a stable personality trait. Individuals high in this trait not only seek to manage aspects of their environment but are also strongly motivated to complete tasks effectively (Ramsey & Etcheverry, 2013). However, when they perceive a lack of control, they may then exhibit behaviours that hinder their performance (Burger, 1985; Ramsey & Etcheverry, 2013; Shapiro, Schwartz & Astin, 1996). Applied to our context, we hypothesize that individuals with a high need for control – as a *trait* characteristic – experience a stronger sense of presence in low-anxiety environments. Their motivation to engage fully may enhance their sense of presence. In the present study, participants were exposed to a non-anxiety-inducing virtual environment, which likely supported their immersion. As a result, no overt control-related behaviours were observed, and their sense of presence remained high – possibly driven by a desire to perform well within the environment. Conversely, in more anxiety-inducing settings, perceived control may be negatively impacted. In such cases, individuals with a strong desire for control might react with behaviours detrimental to their performance and, consequently, their sense of presence, as suggested by Wallach and colleagues (2009).

**Figure 1:**
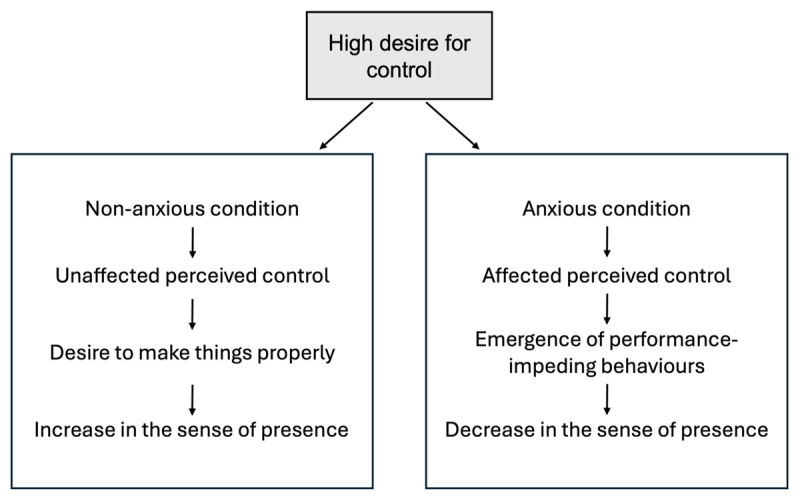
Theoretical model of the impact of the need for control on the sense of presence.

Based on this reasoning, we developed a hypothetical theorical model (Figure 1) to illustrate the impact of a high desire for control on the sense of presence, depending on the condition of immersion.

In the present study, users reported an average sense of presence during immersion. This may be attributed to the statistically significant increase in cybersickness symptoms observed post-immersion (Weech, Kenny & Barnette-Cowan, 2019). However, the scores did not indicate a significant occurrence of cybersickness, as they remained low, but rather the presence of cybersickness symptoms. Despite the increase in cybersickness symptoms, the impact of a strong desire for control on sense of presence was noticeable. This suggests that other variables may play a role in the current theoretical model, and the influence of a strong desire for control on the sense of presence may depend on these additional factors.

### Limitations and experimental and clinical perspectives

Our results should be interpreted considering several limitations.

Given the challenging nature of conceptualizing the need for control, it is possible that the variables used may not comprehensively capture this need in individuals. Future studies should replicate our design using a more specific Desire for Control Scale. Furthermore, it would be valuable to investigate both the desire for control and the sense of control, as the expression of the need for control likely fluctuates during immersion based on the user’s perceived sense of control. This highlights the necessity of assessing the situational expression of the need for control. In the current study, only general tendencies such as perfectionism, intolerance of uncertainty, and avoidance were assessed, without delving into the manifestation of specific behaviours. For instance, future studies would benefit from evaluating distress caused by uncertainty rather than focusing solely on intolerance of uncertainty.

Additionally, future studies should investigate the relationship between the need for control and the sense of presence by varying the immersion condition based on its level of anxiety-inducing characteristics and by applying moderation analyses. Indeed, in the current study, only non-anxiety inducing virtual environments have been tested which limit severely the extent of the results obtained and the forthcoming theorical model. This model is innovative and should be further refined and expanded by incorporating additional variables to better understand the impact of a strong desire for control.

From a practical therapeutic perspective, assessing the need for control prior to immersion in VR could offer significant benefits. Specifically, tailoring VR experiences based on the intensity of an individual’s need for control could enhance their therapeutic effectiveness. For individuals with a high need for control, adopting a gradual and progressive immersion strategy, where the level of challenge or anxiety is carefully increased over time, could enhance their engagement and active participation during the immersive experience.

Several methodological limitations should be acknowledged. First, the power analysis was conducted assuming a large effect size. While this approach can be justified in exploratory contexts, it would be more appropriate to replicate the analysis using a medium effect size, which is generally considered more realistic in psychological research. The relatively small sample size may have limited the ability to capture the full spectrum of the need for control, thereby restricting the generalizability of the findings to the broader population. Moreover, the small sample increases the risk of both Type I and Type II errors. This issue, compounded by a high number of statistical tests conducted, has potentially exacerbated this phenomenon. Additionally, the use of non-parametric tests – while appropriate given distributional assumptions – may have reduced the statistical power to detect significant effects. Future research should address these limitations by employing larger sample sizes, as well as more statistically robust methods. Additionally, future research should test the hypothesis by applying a path model to understand better the functioning of the theoretical model.

## Conclusion

The desire for control appears to influence the sense of presence experienced during immersion in VR. However, its impact would vary depending on the immersive conditions. In an anxiety-inducing condition, the sense of presence could tend to decrease, whereas in a non-anxiety-inducing condition, it would tend to increase. This paper also discussed the experimental implications for further research and the practical therapeutic perspectives.

## Data Accessibility Statement

Data are available upon request from the corresponding author.
